# Measuring Quality of Maternal, Neonatal, Child, Reproductive Health and Nutrition Care with tools developed by the RADAR project and tested in Sub Saharan Africa

**DOI:** 10.1080/16549716.2021.2006469

**Published:** 2022-09-13

**Authors:** Melissa A. Marx, Emily Frost, Elizabeth Hazel, Diwakar Mohan

**Affiliations:** Institute for International Programs, Department of International Health, Johns Hopkins Bloomberg School of Public Health, Baltimore, MD, USA

**Keywords:** Measurement, Direct observation, Simulated clients, Clinical Vignettes, Exit interviews, Program Evaluation, observed structured clinical examinations

## Abstract

Increasing coverage of evidence-based maternal, neonatal, child, reproductive health and nutrition (MNCRHN) programs in low- and middle-income countries has coincided with dramatic improvements in health despite variable quality of implementation. Comprehensive evaluation to inform program improvement requires standardized but adaptable tools, which the Real Accountability, Data Analysis for Results (RADAR) project has developed. To inform selection of tools and methods packages (‘packages’) to measure program quality of care (QoC), we documented experiences testing the packages, which were developed and adapted based on global and local expertise, and pre- and pilot-testing. We conducted cross-sectional studies in 2018–2019 on the quality of 1) integrated community case management, 2) counseling on maternal, infant, and young child feeding, 3) intrapartum care, and 4) family planning counseling in Mali, Mozambique, Tanzania, and Malawi. Herein we describe package performance and highlight experiences that inform their selection and use. Direct observation packages provided high-quality, immediately applicable results but they required specialized expertise, in-person collection, adequate patient volume, reasonable wait times, and unambiguously ‘correct’ provision of care. General satisfaction questions from exit interview packages produced unvaryingly positive responses despite variable observed quality of care. Variation increased when questions were more targeted, but findings on caregiver and client’s recall of recommendations were more actionable. When interactive, clinical vignettes can capture knowledge of clinical care. But for conditions that can be simulated, like provision of family planning counseling, we could capture provider practice from simulated clients. Clinicians could more easily demonstrate tactile aspects of intrapartum care using observed structured clinical examinations, but this method required storage and transport of the required mannequins. Based on our findings we recommend ten questions upon which evaluators can base package selection. Findings from these packages inform programs and, in the context of comprehensive program evaluation enable us to link programs with impact.

## Background

The coverage of life-saving maternal, neonatal, child, reproductive health and nutrition (MNCRHN) programs has increased dramatically in the past 20 years, resulting in dramatic reductions in under-five mortality and improvements in maternal health outcomes globally [1,2]. Emerging evidence suggests, however, that sub-optimal quality of implementation, and in particular, weak technical skill and competency have muted the effect of these investments [3–6].

Despite recent attention on quality of care evaluation [3], the global community continues to *miss chances* to use these data to make informed choices to 1) stop funding programs that fail to improve health, 2) identify and scale up programs that work, and 3) increase support for programs that have potential but lack sufficient long-term investment to operate at a high enough quality to impact health [3,4,7].

Findings from rigorous evaluations using standardized tools and methods can inform these decisions and answer questions like: ‘Is the program being implemented as planned, and at sufficient quality to achieve expected impact?’ And the other four evaluation questions addressed by Real Accountability, Data Analysis for Results (RADAR) project (https://www.radar-project.org) and described by Amouzou, et al. [8].

Knowing what works informs mid-course program correction and guides practice and policy so that resources are focused on developing and maintaining high-quality programs. In the context of comprehensive program evaluations that answer all five questions and consider temporal and/or counterfactual comparisons, quality of care findings can strengthen the level of inference that impacts observed were caused by the program [9].

For RADAR, we developed and disseminated tool and methods packages (‘packages’) that standardize and increase the rigor and comprehensiveness of program evaluation. These packages were tested in the field alongside innovations that aim to increase the efficiency of quality of care evaluations. In this paper we describe lessons learned from testing these packages (available at https://www.radar-project.org/isaqoc) and offer guidance based on lessons learned about which packages to use in various circumstances and settings.

## Methods

We developed and tested packages to evaluate the quality of 1) integrated community-based care for the sick child (iCCM), 2) counseling on maternal, infant, and young child feeding (MIYCF counseling), 3) intrapartum care (IPC), and 4) family planning (FP) in Mali, Mozambique, Tanzania, and Malawi. We chose these programs to evaluate in these countries because in three countries, the funder who was supporting evaluation tool development was also supporting the programs and they wished to use the results to improve the programs as well. In the 4^th^ country (Malawi), we had existing relationships that allowed us to address the funder’s priority on evaluating family planning programs. All programs evaluated were implemented by or in collaboration with government health systems.

**Figure 1.**
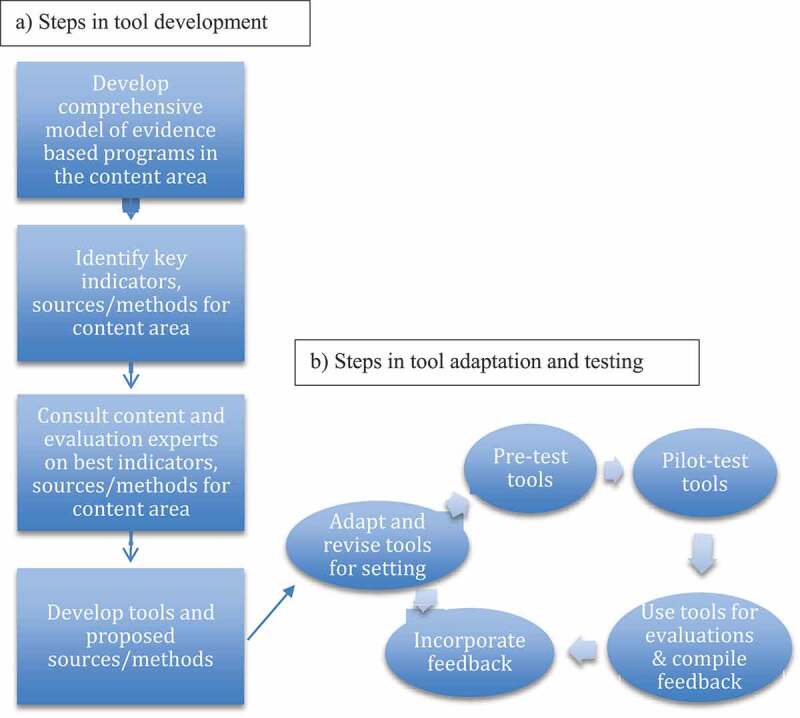
Steps to develop, adapt and test tools.

For each evaluation we undertook a series of steps (Figure 1)

first described and depicted the program to be evaluated in an impact model using a free application: https://evaluationplanningtool.org [10]. To create the data collection tool part of the package, we drew from internationally recognized indicators and instruments [11–13] and consulted global and local experts using on-line surveys and in-person or remote meetings. Local collaborators provided context on each health care system, other similar or complementary programs operating in each program area, and relevant social, epidemiologic, cultural, or geographic factors that might affect the program being evaluated, which informed the adaptation of the indicators, tools, and methods for each evaluation. Methods were chosen to answer globally and locally relevant evaluation questions as part of cross-sectional surveys. Local experts and collaborating providers reviewed and pre-tested the tools, then piloted the packages. All data collection was conducted by trained local teams in the regions’ most commonly spoken languages. All evaluations were approved by both Johns Hopkins School of Public Health’s Institutional Review Board and respective local Research Ethics Boards. All participating health care workers and volunteers (HCW/HCV), beneficiaries, clients and caregivers provided informed consent. The packages and countries in which we tested them are listed in Table 1. We present illustrative results that we think best illustrate the strengths and weaknesses of the packages in various settings and situations. Full sets of methods and results are available at https://www.radar-project.org/isaqoc.

**Table 1.** Tool and methods packages tested (T) and included (I) in the final package and findings from comparisons of methodological innovations, RADAR project.

Topic, country, year of testing		iCCM (Mali, 2018)	FP (Malawi, 2018)	MIYCF counseling (Mozambique, 2019)	Intrapartum Care (Tanzania, 2019)	Experiences
Knowledge	Structured Interviews with providers	T, I	T, I	T	T, I	Mostly used to measure implementation strength
	Clinical vignettes (ClV)	T	T, I		T, I	Performance on tactile and interactive aspects that may not reflect lack of knowledge
Skills	Objective Structured Clinical Examination (OSCE)				T, I	Captured tactile skills and techniques; Some clinicians feared repercussions of poor performance; Time consuming
Provision	Direct observation (DO)	T, I	T, I	T	T, I	Required travel to clinical sites; Time consuming
	Simulated client (SC)		T, I			Performed well on chosen scenarios that did not require invasive procedures
Outcome	Exit interview (EI)	T, I	T, I	T		Very little variation in satisfaction reported; useful data on understanding instructions
Innovations tested			ClV by phone vs. SC	In-person collection of DO vs. from AV recording read by non-expert vs. AV read by expert	ClV vs. OSCE	
Innovation findings			Clinical interactions caught more in SC [22]	Not yet completed*	Similarities but tactile clinical interactions more readily shown than verbalized [21]	
Recommendations			Acceptable substitute for some domains	Not yet completed*	Acceptable substitute for some domains	

iCCM = integrated Community Case Management

FP = Family Planning

ClV = Clinical Vignettes

DO = Direct Observation

SC = Simulated Client

EI = Exit Interviews

AV = Audio Visual

OSCE = Objective Structured Clinical Examinations

**Table 2.** Proportion of beneficiaries demonstrating **correct knowledge** about nutrition topics covered by CHV interview, by beneficiary type *exit interview*, Mozambique 2019.

Topic about which beneficiaries provides the correct answer(s)*	n	%
Pregnant women only (N = 38)		
Foods pregnant women should eat	34	89.5
Services women receive at ANC visits	35	92.1
Women should start breastfeeding within 1 hour of delivery*	24	68.2
Colostrum is good for the baby*	26	68.4
Benefits of colostrum	10	26.3
Pregnant women and women 0–5 months post-partum (N = 69)		
Benefits of breastfeeding	30	43.5
Exclusive breastfeeding should occur for at least the first 6 months	46	66.7
Women 0–5 months postpartum (N = 31)		
Correct signs of a good latch	10	32.3
Women 0–5 months and 6–12 months postpartum (N = 78)		
Foods that is good for babies to eat after 6 months	61	78.2
Women 6–12 months postpartum (N = 47)		
Family planning methods	40	85.1
Benefits of family planning	27	57.5
All beneficiaries (N = 116)		
Ways to prevent diarrhea	74	63.8
Situations for which a person should wash his/her hands	102	87.9

*The correct answer when there is one or at least 2 correct examples when examples are requested

We also explored innovations to increase evaluation efficiency and report excerpts of findings comparing: 1) observed structured clinical examination (OSCE) vs. clinical vignette (ClV) for IPC and 2) phone vs. in-person ClV for FP.

Local and international researchers, program implementers and government officials analyzed data in workshops where the teams prepared: 1) a report, 2) a presentation for local and national stakeholders including Ministry of Health officials and program implementers, and 3) policy brief(s) highlighting key findings and recommendations for local and national policy makers (all available at https://www.radar-project.org/isaqoc).

### Implementation-specific methods and tools

#### Intrapartum care

We designed and tested our **quality of Intrapartum Care** package to assess provider knowledge, skill, and practice for a program to improve Basic Emergency Obstetric and Newborn Care (BEmONC) signal functions in all four health centers and 20 rural health facilities (‘dispensaries’) where the program was implemented in the Simiyu region of Tanzania

Tools include: 1) **provider sociodemographic interview,** 2) **checklists for study clinicians to record** clinician’s procedures *during* direct observation, 3) **OSCEs** and three MamaNatalie mannequins [14] (one per facility at any one time) and 5) **CIVs.** We also developed four clinical scenarios, used for both OSCEs and ClVs: normal delivery with essential newborn care, pre-eclampsia, post-partum hemorrhage and neonatal resuscitation.

#### Data collection methods

We invited all clinicians in program facilities providing intrapartum care to participate. During the direct observations at the health centers, the evaluation clinicians waited for a birth, observed labor and delivery, and recorded observations on the checklist. For OSCEs and ClVs, the evaluation clinicians guided facility providers through each scenario and recorded clinical procedures demonstrated or described, respectively. Providers were randomized on the order of their OSCE and ClV evaluations.

#### Integrated community case management

Our **iCCM tools** were developed and tested to evaluate a program to improve maternal and child health through strengthening iCCM services, among others provided by CHWs in six rural districts of the Koulikoro and Sikasso regions of Mali.

Tools include: 1) **structured sociodemographic and knowledge interviews, 2)** three scenarios and checklists to record responses to **clinical vignettes**, 3) **direct observation and re-examination checklists** of sick children aged 2–59 months, and 4) **caregiver exit interviews**.

#### Data collection methods

All 441 sites with program CHWs were eligible, and 300 CHW sites were selected using stratified random sampling proportional to number of CHW sites per district. Study teams, which included a clinician, visited each site, and interviewed the CHWs and observed two clinical consultations with sick children aged 2–59 months. If fewer than two sick children spontaneously presented, sick children were sought in their homes, which were selected according to a protocol of walking 1 km and selecting the first house on the right. After each consultation, a study team member conducted an exit interview with the caregiver, after which a study clinician re-examined the child and discussed the diagnosis and treatment with the CHW. Caregivers reported on their recall of the CHW’s treatment and care instructions and their satisfaction with the clinical interaction denoting increasing satisfaction with an increasing number of chickens from 1–5.

#### Family planning

We tested family planning tools while evaluating the quality of FP counseling care delivered in government health facilities in rural Malawi. The evaluation aimed to determine the association between quality of FP counseling and care and outcomes such as modern contraceptive prevalence rates.

Tools include: 1) **structured interviews** with clinic-based and community health workers, 2) checklists for **clinical vignettes** 3), **simulated mystery client interactions** (for facility-based providers), 4) and **direct observations** (in ‘high volume’ facilities with >30 FP clients/month), and 5) **client exit interviews. Two clinical scenarios** were used for simulated mystery clients: (1) an adolescent, unmarried woman seeking contraceptive pills, and (2) an adult married woman switching methods to contraceptive pills. These scenarios, plus the following additional scenario were used for clinical vignettes: (3) an adult first-time user of modern contraceptives with a complex medical history.

#### Data collection methods

The survey was conducted of 542 HCWs in 112 facilities in six districts in Malawi, which were purposefully selected to be similar with respect to percent rural/urban, women’s education, religion, facilities per population, and poverty levels and to represent the high (N = 3) and low (N = 3) FP outcomes based on total fertility rates, modern contraceptive prevalence rates, unmet need for modern contraceptives, and proportion with demand for FP services satisfied from the 2016 Demographic Health Survey [15]. All selected facility and community-based HCWs were called before the visit, which they were informed would occur within a 3-month period. In that period, data collection staff visited the providers and collected data. In health facilities, trained simulated clients presented case scenarios first. After their clinical interaction, they debriefed with a supervisor, who completed the checklist. The supervisor then returned to the facility, interviewed the provider who saw the simulated client and in high volume facilities, observed and recorded observations of 1–5 client-consultations. In these facilities another study team member interviewed clients using the exit interview tool and Audio-Computer Assisted Self-Interviewing methods on which clients expressed increasing satisfaction with increasing numbers of chickens, from 1–3. Separately, interviewers called providers on their mobile phones, presented ClV scenarios to them, and recorded responses as with the in-person version.

#### MIYCF counseling (as part of a larger MIYCF program)

We evaluated a CHV-administered MIYCF counseling program provided to beneficiaries who were pregnant or 0–23 months post-partum women at high risk of malnutrition in southern Mozambique.

Tools include: 1) **structured interviews** with CHVs, 2) CHV **knowledge assessments**, 3) **checklists** for **direct observations** and 4) **client exit interviews.**

#### Data collection methods

The survey was conducted among a stratified random sample of 152 of the 329 CHVs working in the two districts in Inhambane province where the program operated. Data collectors conducted the CHV interview and knowledge assessment first. They then observed the CHV counseling the beneficiary and recorded it on the checklist and by using a body camera with built-in microphone. Finally, the beneficiary was interviewed to assess satisfaction with the counseling session and recall of the contents of the session.

To illustrate how these tools have been and can be used to improve practice, we present an example of findings using at least one tool from each implementation (Boxes and Tables), lessons learned from using the tools and an indication of whether the tools were included in the final version (Table 1).

## Results

### Direct observation packages

**Figure 2.**
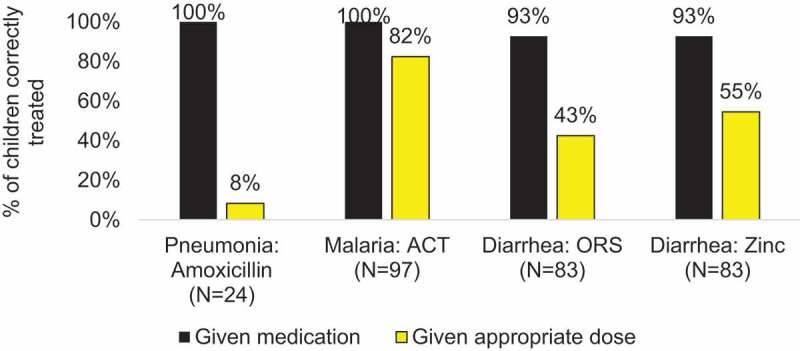
Proportion CHWs providing iCCM services in rural Mali who provided the children they had correctly diagnosed with the correct treatment and dose of medication.

The Direct Observation (DO) tool was developed and tested for all four types of MNCRHN programs (Table 1). As expected, it was the most resource-intensive to implement, requiring clinically trained evaluators for every implementation except for the MIYCF counseling program evaluation. But when it was feasible, it provided actionable evidence of provider practice (Figure 2 and Box 1).

**BOX 1.** 

*Most (83%) children correctly diagnosed with malaria by the CHW providing iCCM care in Mali were given the correct dose of antimarials, but only 13% of children diagnosed with pneumonia were given the correct dose of amoxicillin. And of children diagnosed with diarrhea, 44% were given the correct dose of oral rehydration solution and 3% were given the correct dose of zinc*.

**BOX 2.** 

In Mali, there was little variation in reported satisfaction with the CHW-provided iCCM treatment and care. *A large majority (86%) of care givers who brought a sick child in for evaluation and receiving newborn home visits (91%) said they were satisfied with the care they received*.Similarly, in Malawi, despite variation in measured quality of family planning counseling and care, including documented disrespectful treatment*, clients reported high levels of satisfaction; 80% felt the provider listened and responded to them, 83% said they were well treated by provider and staff*.

**BOX 3.** 

*About half of MIYCF counseling beneficiaries reported learning no new nutrition information from the CHV, and most said the CHV spent just enough time at the counselling session.Almost all said they can follow the CHV’s nutrition advice using food they had in their home*.

**BOX 5.** 

*More than seventy percent of clinicians reported correct management of all measured aspects of third stage labor (71-81%), except only 64% reported that they would deliver the placenta slowly with both hands, turning the placenta, and 57% reported they would massage the uterus until firm after the placenta delivers*.

**BOX 4.** 

*A minority of MIYCF counseling beneficiaries in Mozambique recalled the benefits of colostrum, signs of a good latch, benefits of breastfeeding, and family planning methods*.

**BOX 6.** 

*A very small percentage of family planning providers conducting the initial assessment asked essential questions. Over 63% counseled on how to use oral contraceptive pills, and 61% of adolescent clients received counseling on side effects, a minority of adult clients received counseling on side effects and less than 40% of both types of clients were counseled on whether the method protects against HIV and STI*.

An unstable security situation and flooding in some districts of rural Mali precluded access to some sites and use of the DO package. Also in Mali, many cases, fewer than the protocol-designated two sick children spontaneously presented for care on the day of the evaluation. Our strategy to systematically choose houses from which to seek sick children was impractical because of how households were spread throughout villages. So, we adopted less systematic, but more flexible approaches to finding sick children at home. Before combining DO findings from sick children sought at home and children spontaneously presenting, we stratified analyses to identify any differences between the two groups.

While we wanted to evaluate quality of FP and IPC counseling and care in the community in addition to at all levels of facilities, the volume of family planning services provided was not sufficient in lower-level and community-based care settings to make direct observation efficient. For IPC, even when limiting DOs to the health facilities with the highest volume of deliveries, excluding the time waiting for women to present, each DO required a long wait for labor and delivery: a median of 4.9 hours.

The MIYCF counseling program evaluation relied on a community health volunteer to counsel pregnant and post-partum beneficiaries on local foods to eat and avoid for both themselves and young children. For this DO we captured and categorized each food listed as appropriate to eat or to avoid, which required local expertise. To the evaluation team, the categorization seemed subjective. Further, objectively categorizing each item that was not on these lists and determining how many correct foods that had to be chosen and how to also capture when incorrect foods were also mentioned was cumbersome and seemed both arbitrary and subjective.

### Exit interview packages

Clients and newborn/child caregivers were asked to participate in exit interviews for all evaluations except IPC (which we deemed too burdensome for the woman who just delivered). Our efforts to provide clients locally relevant ways to quantify satisfaction and to minimize biased reporting due to social-desirability and/or provider-client power dynamics in the FP and iCCM implementations (ACASI in Malawi, quantifying satisfaction with chickens in Mali and Malawi) did not appear to improve responses to satisfaction questions that were too generic and broad, as shown in Box 2.

After those implementations, we conducted formative qualitative research in Mozambique to inform and develop quantitative satisfaction questions in the MIYCF counseling tool. Despite these improvements, satisfaction findings proved only marginally useful for informing improvements in programming (Box 3).

All exit interview tools also included questions on caregiver/client knowledge and understanding of recommendations, which provided more useful and actionable data in all three implementations of the exit interview tool package, shown for beneficiary knowledge of nutrition practices in Table 2 and Box 4.

### Clinical vignette packages

Clinical vignettes were used to evaluate FP, iCCM, and IPC programs. The ClV package for iCCM included a brief description of the scenario and a checklist, but no prompts to ascertain the provider’s progress in the clinical interaction. While we had pre-identified the ‘correct’ responses, because there were multiple steps, coding an entire set of actions as correct/incorrect required somewhat arbitrary decisions about the most important items the provider might spontaneously mention (and/or omit). The resulting data were difficult to analyze and interpret.

After this experience we added prompts and used common scenarios for the package for the IPC ClV resulting in data that were easier to analyze and interpret (Box 5). But it took hours to collect data from the three ClVs scenarios. In addition to the interview, OCSE and DO, data collection resulted in substantial missed clinic time and fatigue. In addition, after collecting data in several clinics, we found some providers in clinics visited later using cheat sheets designed to help them ‘do well’ on the evaluation.

Findings from comparing the reliability and validity of results from the ClV to other methods, such as OCSE (IPC) or SC (FP) were the useful for making decisions about which packages to use. But, because two of the three iCCM scenarios relied upon a child having a danger sign, which is rare in real life, we did not have enough real-life instances for a meaningful comparison with the DO in Mali.

### OSCE package

The OSCE package was paired with birthing simulator mannequins [14] and used for assessment of IPC. OSCEs/mannequins provided clinicians a chance to demonstrate procedures they were describing, which likely stimulated their recall of manual procedures, such as physical examinations. In addition to the time burden noted in the previous section, key challenges of using the OSCE tool/method package included obtaining and transporting the mannequins within country and finding a separate and private space within the clinic premises to administer the OCSE. In addition, we sensed that the mannequins may have been intimidating.

When comparing OSCEs and ClVs, we found high agreement for some practices and low agreement on others (Table 1), with few patterns noted – although in some cases more manual procedures, including those demonstrated on the mannequin during third stage of labor (Table 3) were better captured on OSCE than the ClV.

**Table 3.** Comparison of the percent of clinicians reporting the procedures they would do during third-stage labor in response to clinical vignette and OCSE tools, Tanzania.

Essential procedure (N = 77)	Percent reporting essential procedure		
	**ClV**	**OSCE**	**Kappa**
Apply counter traction in upward direction	81	84	0.50
Hold firm and steady tension with cord	74	79	0.68
Deliver placenta slowly with both hands, turning placenta	64	75	0.61
Massage uterus until firm after placenta delivers	57	73	0.37
Examine the vulva, perineum, and vagina for tears	71	86	0.66

**Table 4.** Percent of family planning providers whose counseling and care included the following activities, by case scenario, Malawi.

	SC Scenario	
	Adult (n = 111) %	Adolescent (n = 111) %
Percent of providers who asked client about		
Age	30	32
Desire for more children	5	5
Last menstrual cycle	12	22
Any chronic illnesses	3	8
Conducted ≥1 physical exam*	22	23
Counseled on pill use for clients given pills**		
How to use OCP	63***	87***
Common side effects of OCP	33***	61***
Indicates no HIV/STIs protection from OCP	21***	40***

*Examples: measure blood pressure, weight, check for anemia

**N = 76 Adults; N = 77 Adolescents

***statistically significantly different at p ≤ 0.01

**BOX 7.** Key questions to consider when using the tool packages and brief guidance based on answers.

Key questions	Recommendations based on answers
What is the evaluation question? How will findings be used?	If to guide retraining and service is not life-saving, could choose any package including ClV. If to increase level of causal inference, choose DO or SC.
(2) How locally culturally defined is the program being implemented?	If highly locally defined, package may evaluate fidelity only. Must decide if fidelity is in line with evaluation question.
(3) How valid do you need the answer to be?	If the care service is lifesaving, choose DO or SC for higher validity.
(4) How many and what kinds of resources are available? How much time do you have?	If evaluation is well-supported and time is not limited, choose DO and/or SC.
(5) How frequently is the program provided to beneficiaries in your setting(s)?	If volume of provision of the service is very low, choose ClV and/or OSCE.
(6) Can the health need or requested service be acted?	If it can be acted, consider SC. If not consider DO, ClV and/or OSCE.
(7) Are most or all potential beneficiaries known to the program provider?	If the setting is small and/or beneficiaries are known, avoid SC and choose DO, ClV and/or OSCE.
(8) Can evaluators reach the areas where the program is implemented?	If the area is inaccessible, choose cellphone based ClV.
(9) How reliable is cellular phone service to providers in the program areas?	If cellphone service is not reliable, choose DO, SC, in-person ClV and/or OSCE. If reliable, choose cellphone-based ClV.
(10) Is the evaluation limited to quantitative methods?	If limited, restrict EI questions to ascertaining recall of messages and instructions and avoid attempting to elicit the level of satisfaction with services.

ClV = Clinical Vignettes

DO = Direct Observation

SC = Simulated Client

EI = Exit Interviews

OSCE = Objective Structured Clinical Examinations

### Simulated client packages

We tested SC packages only for FP because it was not possible to simulate labor, practical to ask a child to simulate a childhood illness or feasible to act as a community member in a setting where everyone knows their neighbors. For this evaluation, the SC actors had to adopt scenarios that allowed them to avoid blood draws, injection and/or vaginal exams. This restriction limited the FP methods about which we could evaluate the quality of counseling and care to condoms and contraceptive pills, and we chose contraceptive pills. Regardless, the findings proved actionable for program implementers.

We documented a higher percent of family planning providers knowing to ask specific questions in the ClV but a lower percent of them asking those questions on the SCs. Also, providers said in the ClV that they would recommend more clinical assessments than they did in the SC. Counseling was comparable across the two methods of evaluation (Table 4, Box 6).

## Discussion

Overall, our tests and comparisons showed us that while RADAR’s Quality of Care tool packages are meant to be readily adaptable, they must be chosen and tailored for the specific setting, topic, context, and objectives of each evaluation. While our work is limited by our having tested them exclusively in Sub-Saharan African settings, on MNCRHN programs, and in imperfect data collection contexts (such as in rainy season and translated into local languages), we believe that our experience and the lessons we learned can inform improved quality of care assessments. Based on these lessons, we recommend that the design and tools be informed by evaluators’ answers to ten questions (Box 7).

To guide the choice of tools and methods, evaluators and stakeholders must first agree on the key evaluation questions, objectives, and the intended use of findings (Question 1). For instance, can decisions or inferences be made using findings on knowledge and skill, or is direct observation of practice necessary? How will the data be used? If findings will guide the retraining or scaling up of training of providers, ClV could suffice for specific aspects of some kinds of care, and OSCEs provide even more useful data if mannequins and transport for them are available. But DO and SC provide the gold standard and likely more consistent and valid results. In Mali, findings from the ClV were not easily interpreted. But findings from the DO were useful for program implementers to improve supervision, supervision tools and job aids for the CHWs [16].

Next, it is important to know (Question 2) whether the program is highly locally defined – based on local culture, food (such as our MIYCF counseling program) or available resources, or more universal. If the former, it is important to determine whether the focus of the evaluation is on fidelity or quality. Fidelity can be useful to program implementers but may not contribute evidence to a comprehensive impact evaluation, especially if the program is not evidence-based and/or is outside the causal chain. The inherently locally context-specific nature of dietary recommendations makes developing a standard, adaptable direct observation toolkit to evaluate MIYCF counseling challenging. Specific recommendations must be based on foods that are readily available and preferred locally. Adapting international and national evidence-based recommendations to a local setting requires a rich knowledge of the local culture and context, for which local program implementers are best suited. To serve stakeholder needs, our MIYCF counseling tools captured fidelity, which, while useful for the implementers, does not contribute substantively to comprehensive impact evaluations.

Next, an evaluator must decide on Question 3, the desired level validity of the findings. If the program being evaluated depends on high-quality care and the care determines survival or has long-term consequences, it is important that the results are valid, and evaluators should use DO or SC tools, if possible. But the choice also depends on how much time and resources are available (Question 4) and the volume of services provided where the evaluation will take place (Question 5). Direct observation tools are expensive and time consuming; best used when there is a relatively high frequency or volume of the measured event or services; and when there are no physical or safety barriers that preclude reaching the care setting. They are also subject to the Hawthorn effect, where the observed act differently precisely because they are being observed, which has been reported in other studies as well [17]. SC methods are not subject to the Hawthorne effect, but they can only be used for services that can be acted (e.g. not childhood illness or pregnancy; Question 6), when playing the role does not require the actor to submit to unwanted procedures, and where services are being provided to a large enough population that providers or clients do not know all clients normally seeking services there (Question 7). Hormonal injection is the most used contraception in Malawi, but scenarios had our SC clients asking for hormonal pills instead, so our SCs were presenting relevant but not the most common scenarios. Our SCs were instructed to refuse invasive tests such as blood draws/vaginal exams, which may have left SCs in our evaluation open to unmasking. However, we captured instances of disrespectful care, and some instances of abuse [18], so we believe that most if not all of them remained masked. Evaluators must consider these constraints and opportunities. One innovative way that technical quality has been captured in community settings without unmasking SCs is to recruit women from the community that are actually seeking care and train them as ‘mystery shoppers’ [19]. This method could also accommodate evaluations of invasive interactions such as blood draws and vaginal exams. If too many barriers exist to SC, knowledge and skill ascertained using ClVs can provide a reasonable estimate of technical quality [20] and our results show it can be used for FP counseling or IPC (with or without OSCE) in lower-level health facilities and of FP in community settings. ClV can be used to measure quality of care for specific aspects of labor, delivery, newborn and postpartum care when DO and SC are not feasible [21].

For DO, SC and OSCE, the evaluator also must be able to reach the care setting (Question 8). Where areas are inaccessible due to geography or conflict, and cell coverage is sufficient (Question 9), the cellphone-based ClV is a reasonable option to capture skill and knowledge to approximate technical quality [22]. Family planning providers presented with an actual person in their clinical setting seemed to be prompted better recall and practice [22] than those presented with scenarios. Although a less clear pattern was seen from the in-person ClV vs. IPC tool comparison; OSCEs seemed to provide clinicians a chance to demonstrate procedures they were describing, which likely elicited better recall of the more tactile steps in labor, delivery, and newborn/postpartum care. To best approximate technical quality, evaluators should limit the domains they capture with ClVs to those that best demonstrate agreement with SCs or DOs [9,23]. Extensive pre-testing and piloting of cellphone or in-person ClVs is also necessary to ensure the resulting data are useful, readily interpretable and justify the time required to conduct them.

Findings from exit interviews can provide useful information about quality of care as interpreted through the ability of clients to correctly report recommendations and instructions, although they test both the knowledge and the competency of the provider to convey information to the client and must be coupled with knowing what the provider recommended (nearly always with a DO). Other studies suggest even when used to measure client recall, that compared to DOs, exit interviews suffer from low specificity because it is difficult to link all of their knowledge on a subject to one clinical interaction [24,25].

Although some literature suggests that patient satisfaction can inform improvement of the quality of services [26] quantitative exit interview questions assessing satisfaction were difficult to create and findings from them difficult to interpret. Initial satisfaction questions were too broad. Additionally, like in other studies [27,28] they may have elicited socially desirable responses, perhaps reflecting asymmetric power between clients and their providers as previous studies have shown [29], and clients’ low expectations for the quality of their care. Our attempts to shield answers from the providers and the evaluators (latter using ACASI) did not result in more variation in results. And our attempts to quantifying satisfaction pictorially – using chickens – was confusing for the clients/caregivers, who remained satisfied with services despite low measured technical quality. Our qualitative work allowed us to improve the specificity of our third set of questions, but results were still of marginal use for improving the program. The qualitative findings were more enlightening, so in light of the myriad advantages of qualitative and mixed methods studies [30] we recommend when possible (Question 10) collecting client satisfaction qualitatively instead of quantitatively.

The experiences described herein provide insights into how findings from evaluations using these tools can inform program improvement and contribute evidence to comprehensive impact evaluations. They can also help implementers decide how to best collect and use data to make decisions about their programs. The main objective of this paper was to share our lessons learned to guide evaluators to choose appropriate tool packages. We have also, where possible, compared our lessons and experiences to those found in the published literature, but because of the complexity of conveying results of multiple comparisons in one paper and the secondary nature of this objective, we have selected only a small subset of the published literature for comparison.

The guidance and packages presented herein, which include operations manuals as well as sample size calculation and analysis support worksheets are freely available (https://www.radar-project.org/isaqoc). With these packages, instead of creating them, evaluators wishing to answer locally relevant and actionable program evaluation questions can select their tools and methods, adapt them for their country and health system context, and prepare for use. Findings from carefully designed and rigorously executed quality of care studies can identify areas of weak program implementation and augment the level of causal inference as part of a comprehensive program evaluation, ultimately raising the bar on quality, and leading to improved health and survival.

## Paper context

Variable implementation of evidence-based maternal child health programs has limited their impact, and some quality of care evaluation has lacked rigor. We developed and tested quality of care evaluation tool packages in four sub–Saharan African countries and describe experiences to inform use. Based on findings we recommend using ten questions to guide package selection. These freely available packages form an essential component of comprehensive program evaluation and should be used to inform program improvement.
